# From Exhaustion to Empowerment: A Pilot Study on Motor Control-Based Exercise for Fatigue and Quality of Life in Long COVID-19 Patients

**DOI:** 10.3390/medicina62010210

**Published:** 2026-01-20

**Authors:** Carmen Jiménez-Antona, Ricardo Moreta-Fuentes, David Varillas-Delgado, César Moreta-Fuentes, Sofía Laguarta-Val

**Affiliations:** 1 Department of Physical Therapy, Occupational Therapy, Rehabilitation and Physical Medicine, Faculty of Health Sciences, Universidad Rey Juan Carlos (URJC), 28922 Madrid, Spain; carmen.jimenez@urjc.es (C.J.-A.);; 2 Fisyos Center, Método Moreta, 28027 Madrid, Spain; metodomoreta@gmail.com (R.M.-F.); mofuce@gmail.com (C.M.-F.); 3 Department of Exercise and Sport Science, Faculty of Health Sciences, Universidad Francisco de Vitoria, Pozuelo, 28223 Madrid, Spain; 4 FREMAP Care Center, 28946 Madrid, Spain; 5 Cognitive Neuroscience, Pain, and Rehabilitation Research Group (NECODOR), Faculty of Health Sciences, Universidad Rey Juan Carlos (URJC), 28922 Madrid, Spain

**Keywords:** Long COVID-19, exercise therapy, rehabilitation, fatigue, body composition, quality of life

## Abstract

*Background and Objectives*: Long COVID-19 (LC) is a multifaceted condition characterized by persistent fatigue and impaired health-related quality of life (HRQoL). Exercise intolerance and post-exertional symptom exacerbation (PESE) pose challenges for rehabilitation. This study aimed to evaluate the effects of a 12-week core-focused plank exercise program on fatigue and HRQoL in women with LC, using validated patient-reported measures. *Materials and Methods*: A pilot quasi-experimental design was implemented, with non-randomized group allocation. Thirty-nine women with LC were recruited from the Madrid Long COVID Association. Participants were assigned to either an intervention group (n = 20), which completed a supervised plank-based motor control program, or a control group (n = 19), which maintained usual activity. Fatigue was assessed using the Modified Fatigue Impact Scale (MFIS), and HRQoL was measured using the EQ-5D-5L and EQ Visual Analog Scale (EQ-VAS). Body composition was evaluated via bioelectrical impedance analysis. *Results*: The intervention group showed significant reductions after intervention in the MFIS total scores compared to the control group, particularly in the physical (21.26 ± 6.76 vs. 25.21 ± 6.06; *p* < 0.001) and psychosocial domains (4.51 ± 0.41 vs. 5.21 ± 0.38; *p* < 0.001), without triggering PESE. EQ-VAS scores improved significantly (63.94 ± 15.33 vs. 46.31 ± 14.74; *p* = 0.034). No significant changes were found in body composition parameters, suggesting that benefits were driven by neuromuscular adaptations rather than morphological changes. *Conclusions*: A core-focused, non-aerobic exercise program effectively reduced fatigue and improved perceived health status in women with LC. These findings support the use of motor control-based interventions as a safe and feasible strategy for LC rehabilitation, particularly in populations vulnerable to PESE, suggesting clinical applicability for the rehabilitation of women with LC. Further randomized trials are warranted to confirm these results and explore long-term outcomes.

## 1. Introduction

Post-acute sequelae of SARS-CoV-2 infection (PASC), commonly known as Long COVID (LC), is a complex and heterogeneous condition that can affect multiple organ systems, including but not limited to the respiratory, cardiovascular, neurological, gastrointestinal, and musculoskeletal systems [1].

Cognitive and respiratory symptoms are among the most prevalent and clinically significant manifestations of LC. Cognitive impairments often include memory difficulties, reduced concentration, and challenges with complex tasks [2,3], collectively described as ‘brain fog,’ which may persist for months post-infection [4]. Neuropsychological assessments reveal deficits in working memory, attention, and executive functioning even after mild illness [5,6,7], frequently accompanied by anxiety, depression, and sleep disorders, further impacting quality of life [8,9]. Proposed mechanisms are multifactorial, involving neuroinflammation, endothelial dysfunction, cerebral hypoxia, and potential autoimmune responses [10].

Respiratory symptoms are equally prominent in LC and include persistent dyspnea, chest tightness, and chronic cough. These symptoms may fluctuate over time and are not always correlated with radiographic or pulmonary function test abnormalities, posing challenges for clinical evaluation and management [11,12,13]. Fatigue, which is closely linked to both respiratory and cognitive dysfunction, has emerged as a hallmark feature of the syndrome. The prevalence of fatigue in patients with LC syndrome is approximately 32% to 71% in several longitudinal cohorts [14,15,16,17,18]. Emerging evidence suggests that viral persistence, immune dysregulation, and disruption of the gut–lung–brain axis may contribute to the chronicity of these symptoms [19,20,21]. Taken together, the multisystemic nature of LC necessitates a comprehensive diagnostic and therapeutic approach, integrating neurocognitive evaluation and respiratory assessment to optimize patient outcomes and guide future research efforts.

Recent evidence underscores the therapeutic potential of structured physical exercise in alleviating core symptoms of LC, particularly fatigue and impaired health-related quality of life (HRQoL). Fatigue remains one of the most prevalent and disabling manifestations of Long COVID, often persisting for months and significantly limiting daily functioning, social participation, and emotional well-being [22]. Exercise intolerance and post-exertional symptom exacerbation (PESE) have raised concerns regarding the safety of physical rehabilitation. However, symptom-titrated and individualized exercise programs have demonstrated favorable outcomes without increasing adverse events [23,24].

The EuroQoL-5D-5L (EQ-5D-5L) instrument has emerged as a robust tool for quantifying HRQoL in LC populations [25]. It evaluates five dimensions—mobility, self-care, usual activities, pain/discomfort, and anxiety/depression. Studies have shown that exercise-based rehabilitation programs can lead to clinically meaningful improvements in EQ-5D-5L index scores and visual analogue scale (VAS) ratings. Improvements are particularly notable in the “mobility” and “usual activities” dimensions, reflecting enhanced functional capacity and autonomy [26,27].

In a multicenter cohort study, LC patients undergoing geriatric rehabilitation exhibited significant gains in EQ-5D-5L scores over a six-month period, independent of baseline frailty status [28]. Similarly, a 12-week blended digital and community-based rehabilitation program yielded improvements in EQ-5D-5L utility scores, alongside reductions in dyspnea and fatigue, and enhanced emotional well-being [29]. These findings support the integration of exercise therapy into LC management strategies, emphasizing the need for personalized, progressive, and symptom-aware protocols. The EQ-5D-5L not only facilitates the monitoring of patient-reported outcomes but also enables cost–utility analyses, essential for healthcare planning.

Fatigue is among the most persistent and disabling symptoms in LC, affecting physical, cognitive, and psychosocial domains. The Modified Fatigue Impact Scale (MFIS) is widely used to assess its multidimensional impact [30]. Evidence indicates that therapeutic exercise interventions, particularly motor control and core-stabilization strategies, can significantly reduce MFIS scores in the LC population [31]. Systematic reviews confirm that individualized, symptom-titrated programs are effective, well-tolerated, and associated with minimal adverse events, improving fatigue, physical function, and HRQoL when PESE is carefully managed. Based on this, personalized, progressive, non-aerobic exercises such as core-focused routines may offer a safe and effective approach for LC patients, especially those with postural instability or autonomic dysfunction [32,33].

Despite these promising findings, significant gaps remain in the scientific understanding of how exercise interventions influence fatigue and HRQoL in individuals with LC. Existing studies are often limited by small sample sizes, heterogeneous methodologies, and short follow-up durations. Moreover, the variability in symptom presentation and the lack of standardized rehabilitation protocols hinder the generalizability of results. Further research and future direction are needed to establish evidence-based guidelines, optimize intervention design, and clarify the mechanisms through which physical activity exerts its therapeutic effects in this population.

Therefore, the main objective of this study was to evaluate the impact of a 12-week core-focused plank exercise program on fatigue and HRQoL in women with persistent LC symptoms. The study aimed to (i) assess changes in fatigue using the MFIS, focusing on its physical, cognitive, and psychosocial domains; (ii) evaluate improvements in HRQoL using the EQ-5D-5L and EQ-VAS instruments, with attention to both global health perception and specific functional dimensions; (iii) determine whether the intervention influenced body composition, including segmental fat and muscle mass, as measured by bioelectrical impedance analysis; and (iv) explore the safety and feasibility of a non-aerobic, motor control-based exercise regimen in a population at risk for PESE.

## 2. Materials and Methods

### 2.1. Design

A pilot quasi-experimental design with non-randomized group allocation was conducted involving women with LC.

This study was conceived as a pilot trial to evaluate feasibility and preliminary efficacy; therefore, no formal sample size calculation was performed. Based on published recommendations, pilot studies typically include 30–50 participants to estimate variability and inform power calculations for future randomized trials [34,35,36]. Using expected effect sizes for fatigue reduction in Long COVID (Cohen’s d ≈ 0.5), a fully powered trial with 80% power and α = 0.05 would require approximately 100–120 participants in total. Our sample of 39 participants therefore falls within the recommended range for pilot studies and should be interpreted as exploratory and hypothesis-generating rather than confirmatory.

### 2.2. Recruitment Subject

Eligible candidates, identified through consecutive non-probability sampling, received detailed study information and documentation via email and were subsequently invited by phone to attend a screening appointment with the Rehabilitation Physician—an attending doctor at Ramón y Cajal University Hospital and part-time faculty member at Rey Juan Carlos University.

Inclusion criteria required participants to have the following characteristics: (i) persistent symptoms consistent with LC for more than one year, primarily fatigue; (ii) aged ≥18 years; and (iii) completion of the full COVID-19 vaccination schedule as defined by the Spanish Ministry of Health. Exclusion criteria included (i) severe cardiovascular disease, (ii) abdominal hernia, (iii) pregnancy, (iv) recent musculoskeletal injury or surgery (within the past year), and (v) the presence of neuromuscular disorders.

In total, 39 women with LC (mean time since infection: 19.74 ± 7.73 months) were recruited between February and April 2022; 20 patients were assigned to the 12-week plank-based strength training program in the intervention group and 19 participants to the control group.

Baseline group equivalence was assessed by comparing demographic and clinical characteristics; differences were examined for potential clinical relevance, and statistical power was considered to ensure adequate comparability (Table 1).

All participants provided written informed consent. The study protocol was approved by the Ethics Committee of the Hospital Universitario Fundación Alcorcón (approval code: 21/173), and the confidentiality of the participants was ensured, complying with the Declaration of Helsinki 1964 (latest update 2013).

### 2.3. Group Allocation

Group allocation was non-randomized; participants who could commit to attending two supervised sessions per week for 12 weeks were assigned to the intervention group, while those unable to commit were placed in the control group. This pragmatic approach was chosen to maximize feasibility under real-world conditions; however, it may introduce selection bias, as individuals with greater availability or motivation could be overrepresented in the intervention group. To mitigate this, baseline comparability between groups was assessed using demographic and clinical characteristics (Table 1), and statistical analyses accounted for observed differences where relevant.

### 2.4. Assessment Procedures

All assessments were conducted at the Faculty of Health Sciences, Rey Juan Carlos University (Spain). Baseline physical data included measurements of height (cm) and weight (kg). Body composition was measured using bioelectrical impedance analysis (Tanita BC-545N). From these values, body mass index (BMI) was calculated as weight divided by height squared (kg/m^2^) and classified according to World Health Organization (WHO) criteria: underweight (<18.5 kg/m^2^), normal weight (18.5–24.9 kg/m^2^), overweight (25.0–29.9 kg/m^2^), and obesity (≥30.0 kg/m^2^) [37]. In addition to BMI, the following body composition variables were recorded. Total body fat percentage (%BF): This indicates the proportion of fat mass relative to total body weight. WHO guidelines suggest healthy ranges vary by age and sex, typically 10–20% for men and 18–28% for women in adults [38]. Additionally, regional body fat percentages were assessed in the upper limbs, lower limbs, and trunk. Total body water percentage (%TBW): This reflects hydration status, with normal values generally ranging from 50 to 65% depending on sex and age [39]. Muscle mass percentage (%MM): This represents the proportion of skeletal muscle relative to total body weight. Although WHO does not provide strict cut-offs, higher values are generally associated with better functional status and metabolic health [40]. All measurements were taken under standardized conditions. Participants were instructed to avoid vigorous physical activity for at least 24 h prior to the assessment and to urinate immediately before the evaluation to ensure measurement reliability. All assessments were performed by two licensed physiotherapists trained in standardized protocols. Prior to data collection, inter-rater reliability was checked through pilot evaluations to ensure consistency and reproducibility of measurements.

Fatigue was assessed in both groups before and after the intervention of the MFIS, a validated 21-item questionnaire widely used in LC research [41,42]. The MFIS evaluates the perceived impact of fatigue over the previous four weeks across three domains: physical (9 items, score range: 0–36 arbitrary units (a.u.)); cognitive (10 items, score range: 0–40 a.u.); and psychosocial functioning (2 items, score range: 0–8 a.u.). The total score ranges from 0 to 84 a.u., with higher scores indicating greater fatigue-related impairment. A total MFIS score of 38 a.u. or higher is generally considered indicative of clinically significant fatigue [43]. Although no psychometric validation of the MFIS version translated into Spanish was found, the original instrument has shown strong internal consistency (α = 0.81) in similar clinical populations [44].

HRQoL was assessed pre- and post-intervention using the EQ-5D-5L, a standardized and validated instrument widely used in clinical and epidemiological research. The EQ-5D-5L comprises a descriptive system covering five dimensions—mobility, self-care, usual activities, pain/discomfort, and anxiety/depression—each rated on five levels of severity. In addition to the descriptive profile, a dimension-based numerical score was calculated by summing the values assigned to each of the five dimensions. This total score ranges from 5 (indicating no problems in any dimension) to 25 (indicating extreme problems in all dimensions). Higher scores reflect a worse overall health status, while lower scores indicate better perceived health [25]. Furthermore, participants rated their overall health using the EQ-VAS, which ranges from 0 a.u. (worst imaginable health) to 100 a.u. (best imaginable health), providing a quantitative measure of self-perceived health status [45]. The Spanish version of the EQ-5D-5L has demonstrated good internal consistency (Cronbach’s α = 0.75, 95% CI 0.64–0.83; domain range 0.63–0.77) in LC populations [25].

### 2.5. Intervention

Participants in the intervention group underwent a core-stabilization program based on plank exercises, designed to enhance muscular strength and neuromuscular control [46]. The intervention aimed to improve key physical attributes such as power, strength, balance, and proprioception [47]. The program was implemented following the MORETA methodology^®^ and was supervised throughout by two licensed physiotherapists to ensure correct execution and safety.

To optimize instruction and individual attention, participants were divided into two groups. The intervention consisted of 60 min sessions, conducted twice weekly over a 12-week period. Each session was structured into three phases: a 10 min warm-up involving dynamic stretching exercises, a 40 min core training segment focused on abdominal and plank-based movements performed without external loads, and a 10 min cool-down phase incorporating breathing techniques, static stretching, and muscle relaxation exercises.

Participants progressively increased their workload throughout the intervention. In the initial sessions, they performed between 150 and 200 sit-ups per class, reaching 300 to 400 repetitions by the end of the program. Each week, more complex exercises were introduced, combining leg or arm movements with abdominal exercises to enhance muscle tone, proprioception, and the acquisition of new motor patterns involved in trunk stabilization.

During the exercises, continuous monitoring was in place. There was one physiotherapist for every 10 people. We instructed participants to stop if they experienced discomfort, slurred speech, or difficulty speaking during exercise. Almost all exercises were performed in a supine position (American Heart Association recommendation). This strategy is recommended for post-COVID athletes returning to sport to avoid orthostatic tachycardia and to better tolerate the return to exercise [48]. No hydration was provided or required for participants during the sessions. Exercise intensity was maintained at a moderately high level throughout the program, as assessed using the Perceived Exertion Rating Category Scale.

Participants assigned to the control group were instructed to maintain their usual daily physical activity routines throughout the 12-week study period. No specific training or intervention was provided for this group. This approach allowed for comparison between the effects of the structured core-stabilization program and general habitual activity on the targeted physical outcomes.

### 2.6. Statistical Analysis

All statistical analyses were performed using IBM SPSS Statistics version 29.0 (IBM Corp., Armonk, NY, USA). Descriptive statistics were used to summarize baseline characteristics, with continuous variables expressed as means and standard deviations, and categorical variables as frequencies and percentages. The Shapiro–Wilk test was applied to assess the normality of continuous data distributions.

To evaluate the effects of the 12-week core-focused plank exercise intervention, a combination of parametric tests was employed. Paired t-tests were used to assess within-group changes in body composition—MFIS, EQ-5D-5L, and EQ-VAS—from pre- to post-intervention. Between-group comparisons were conducted using two-way repeated measures ANOVA (Group × Period), allowing for the detection of main effects and interaction effects. Assumptions for ANOVA were verified prior to analysis, including homogeneity of variance (Levene’s test).

Chi-square tests were used to compare categorical variables across the five EQ-5D-5L dimensions (mobility, self-care, usual activities, pain/discomfort, and anxiety/depression) between groups and time points.

Significance was set at *p* < 0.05.

## 3. Results

Between February and April 2022, individuals meeting the eligibility criteria were identified through consecutive non-probability sampling. They received comprehensive information about the study via email and were subsequently contacted by phone to schedule a screening visit with the Rehabilitation Physician—an attending specialist at Ramón y Cajal University Hospital and adjunct faculty at Rey Juan Carlos University. Ultimately, 39 participants were enrolled; 20 were allocated to the intervention group, which followed a 12-week plank-based strength training program, while the remaining 19 formed the control group.

### 3.1. Body Composition

#### 3.1.1. Weight

**Figure 1:**
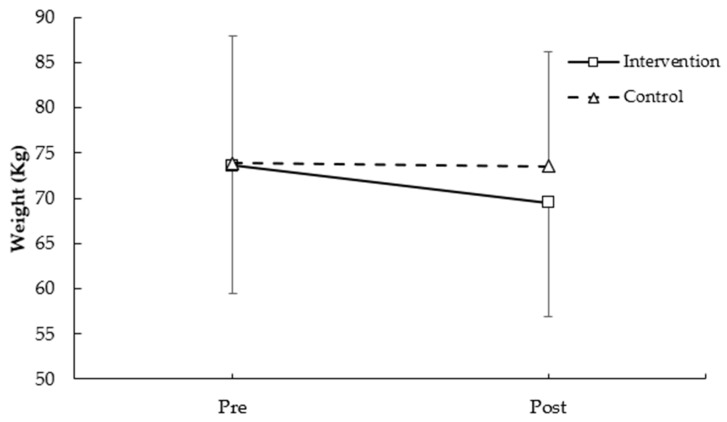
Weight pre- and post-intervention in participants with and without plank-based strength training.

Figure 1 shows weight values before and after the intervention in both groups. No statistically significant differences were observed between groups or across time points (F = 0.291, *p* = 0.597 for group; F = 1.249, *p* = 0.279 for time; group × time: F = 0.786, *p* = 0.388).

#### 3.1.2. Segmental Fat

**Figure 2:**
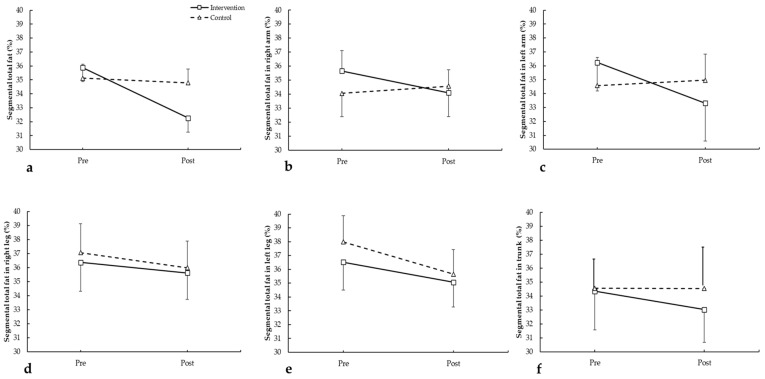
Segmental total fat in arms and legs pre- and post-intervention in participants with and without plank-based strength training. (**a**) Segmental total fat; (**b**) segmental total fat in right arm; (**c**) segmental total fat in eft arm; (**d**) segmental total fat in right leg; (**e**) segmental total fat in left leg; (**f**) segmental total fat in trunk.

For total body fat (Figure 2a), no significant differences were found between groups or over time (F = 0.291, *p* = 0.597 for group; F = 1.249, *p* = 0.279 for time; group × time: F = 0.786, *p* = 0.388). Similarly, for fat in the right arm (Figure 2b), results showed no significant changes (F = 0.028, *p* = 0.870 for group; F = 0.149, *p* = 0.704 for time; group × time: F = 0.690, *p* = 0.417). For the left arm (Figure 2c), no differences were detected (F = 0.000, *p* = 0.999 for group; F = 0.874, *p* = 0.362 for time; group × time: F = 1.565, *p* = 0.227). In the right leg (Figure 2d), results were also non-significant (F = 0.029, *p* = 0.868 for group; F = 0.575, *p* = 0.458 for time; group × time: F = 0.690, *p* = 0.417). For the left leg (Figure 2e), no significant differences were found (F = 0.104, *p* = 0.751 for group; F = 1.842, *p* = 0.191 for time; group × time: F = 0.116, *p* = 0.737). Finally, trunk fat (Figure 2f) showed no significant changes (F = 0.093, *p* = 0.763 for group; F = 0.142, *p* = 0.710 for time; group × time: F = 0.141, *p* = 0.712).

#### 3.1.3. Body Water

**Figure 3:**
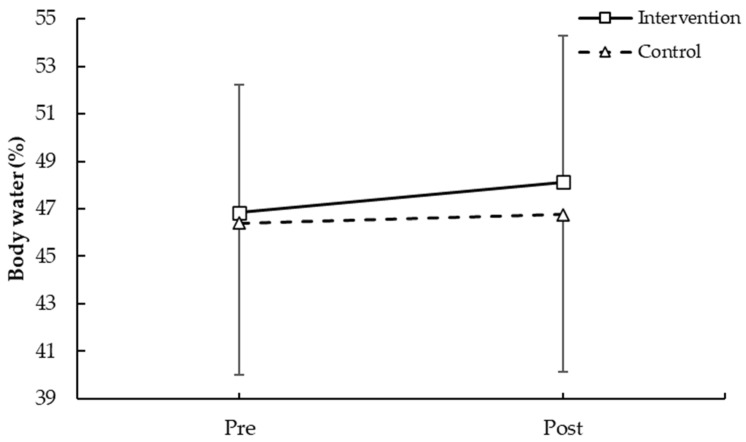
Body water pre- and post-intervention in participants with and without plank-based strength training.

As shown in Figure 3, body water percentage did not differ significantly between groups or across time (F = 0.223, *p* = 0.642 for group; F = 0.850, *p* = 0.369 for time; group × time: F = 0.188, *p* = 0.670).

#### 3.1.4. Segmental Muscle

**Figure 4:**
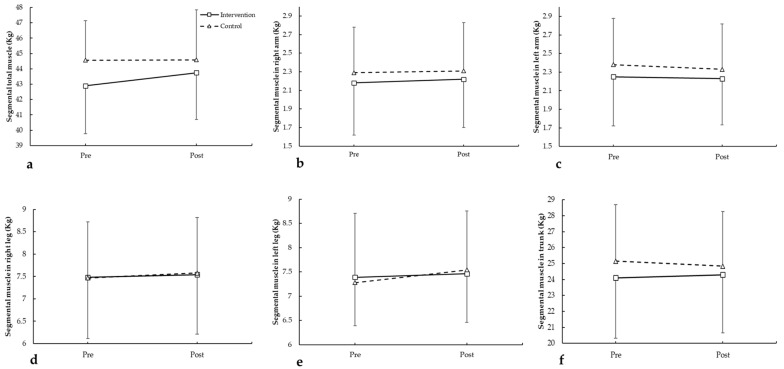
Segmental total muscle in arms and legs pre- and post-intervention in participants with and without plank-based strength training. (**a**) total muscle mass; (**b**) segmental muscle in right arm; (**c**) segmental muscle left arm; (**d**) segmental muscle in right leg; (**e**) segmental muscle in left leg; (**f**) segmental muscle in trunk.

For total muscle mass (Figure 4a), no significant differences were observed (F = 0.369, *p* = 0.551 for group; F = 0.255, *p* = 0.620 for time; group × time: F = 0.257, *p* = 0.619). In the right arm (Figure 4b), results were non-significant (F = 0.386, *p* = 0.542 for group; F = 0.111, *p* = 0.743 for time; group × time: F = 0.086, *p* = 0.773). For the left arm (Figure 4c), no differences were found (F = 0.611, *p* = 0.445 for group; F = 0.385, *p* = 0.542 for time; group × time: F = 0.092, *p* = 0.765). In the right leg (Figure 4d), results showed no significant changes (F = 0.002, *p* = 0.966 for group; F = 0.304, *p* = 0.588 for time; group × time: F = 0.021, *p* = 0.887). For the left leg (Figure 4e), no differences were detected (F = 0.001, *p* = 0.972 for group; F = 1.092, *p* = 0.310 for time; group × time: F = 0.259, *p* = 0.617). Finally, trunk muscle mass (Figure 4f) showed no significant changes (F = 0.569, *p* = 0.460 for group; F = 0.023, *p* = 0.881 for time; group × time: F = 0.440, *p* = 0.515).

### 3.2. Modified Fatigue Impact Scale (MFIS)

**Figure 5:**
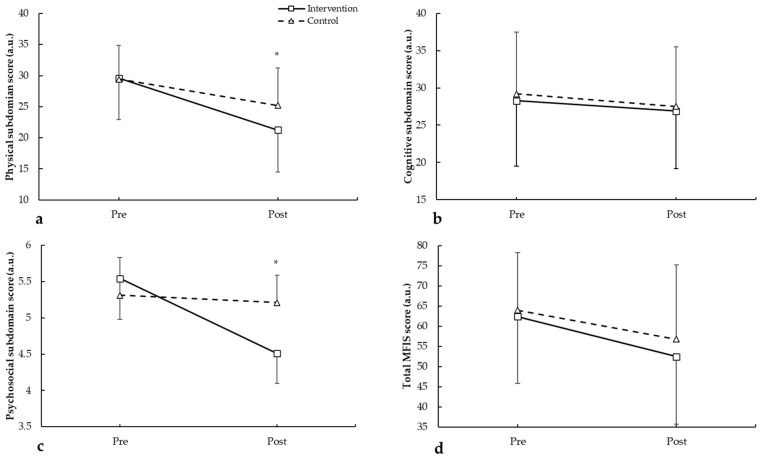
MFIS pre- and post-intervention in participants with and without plank-based strength training, showing the (**a**) physical subdomain, (**b**) cognitive subdomain, (**c**) psychosocial subdomain, and (**d**) total MFIS scores. * *p* < 0.050.

In the physical subdomain (Figure 5a), there was a main effect of the period (F = 39.692, *p* < 0.001), with no main effect of the group (F = 1.229, *p* = 0.282). There was no statistically significant interaction during intervention of group × period (F = 1.882, *p* = 0.187), showing an improvement in the intervention group, 39.1% compared to 16.7% in the control group. For the cognitive subdomain (Figure 5b), there were no main effects of the group (F = 0.086, *p* = 0.773) and period (F = 1.783, *p* = 0.198). There was no statistically significant interaction during intervention in group × period (F = 0.015, *p* = 0.903), with an improvement in the intervention group of 3.5% vs. 3.4% in the control group. Regarding the psychosocial subdomain (Figure 5c), there were no main effects of the group (F = 0.204, *p* = 0.657) and period (F = 2.529, *p* = 0.129). However, there was statistically significant interaction during intervention for group × period (F = 7.489, *p* = 0.014), showing an improvement in the intervention group of 22.2% vs. 5.2% in the control group. Finally, the total MFIS score (Figure 5d) showed main effect of the period (F = 12.427, *p* = 0.002) with no main effect of the group (F = 0.307, *p* = 0.586) and no statistically significant interaction during intervention for group × period (F = 0.369, *p* = 0.551), with an improvement in the intervention group of 14.2% vs. 16.3% in the control group.

### 3.3. EuroQol-5D-5L (EQ-5D-5L)

**Figure 6:**
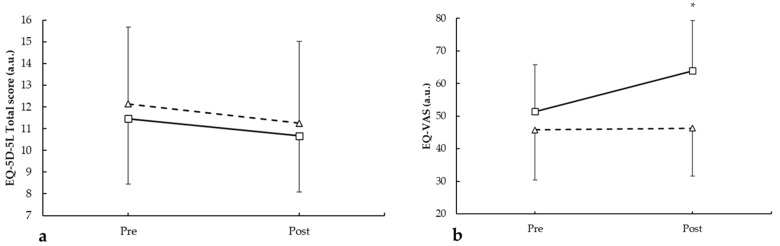
EQ-5D-5L pre- and post-intervention in participants with and without plank-based strength training, showing the (**a**) EQ-5D-5L total score and (**b**) EQ-VAS. * *p* < 0.050.

For the mobility dimension, no differences were found between groups in pre- and post-intervention (*p* = 0.212 and *p* = 0.720, respectively). Regarding the self-care dimension, no differences were found between groups pre- and post-intervention (*p* = 0.420 and *p* = 0.378, respectively). Also, for the usual activities dimension, there was no difference between the groups pre-intervention (*p* = 0.478) and post-intervention (*p* = 0.056). In the pain/discomfort domain, no differences were found between groups in pre- and post-intervention (*p* = 0.089 and *p* = 0.153, respectively). Finally, in the anxiety/depression dimension, pre- and post-intervention showed no differences between the groups (*p* = 0.455 and *p* = 0.536, respectively). However, the total scores (Figure 6a) showed a main effect for the period (F = 5.386, *p* = 0.032) with no main effect for the group (F = 0.362, *p* = 0.555). There was no statistically significant interaction during intervention for group × period (F = 0.011, *p* = 0.919). Regarding the EQ-VAS score (Figure 6b), there were main effects for the period (F = 5.294, *p* = 0.034) and for the group (F = 6.469, *p* = 0.020). There was a statistically significant interaction between the group × period (F = 4.347, *p* = 0.046), with an improvement in the intervention group of 19.6% vs. 1.1% for the control group.

## 4. Discussion

This pilot study suggests that a 12-week core-focused plank exercise program may help reduce fatigue and improve perceived health status in women with LC. The intervention improved the physical and psychosocial domains of the MFIS, reducing scores by 39.1% and 22.2%, respectively, compared to 16.7% and 5.2% in the control group. Although the percentage reduction in total MFIS score was slightly higher in the control group (16.3%) than in the intervention group (14.2%), this difference was not statistically significant and likely reflects variability in a small sample. Notably, the intervention group showed greater improvements in the physical and psychosocial domains, which are clinically relevant dimensions of fatigue. HRQoL, assessed through the EQ-VAS, improved by 19.1% in the intervention group compared to only 1.1% in the control group, indicating enhanced self-perceived health. These findings align with previous research, suggesting that individualized, symptom-aware exercise regimens are both effective and well-tolerated in LC populations [32,49].

Notably, the intervention did not produce significant changes in body composition parameters such as weight, segmental fat, or muscle mass. This suggests that the observed benefits were not mediated by morphological changes but rather by neuromuscular adaptations and improved motor control, likely mediated by neuromuscular adaptations rather than morphological changes. Similar results were reported by Laguarta-Val et al., who found increased muscle activation and reduced fatigue following a plank-based program in LC patients [41]. The intervention’s emphasis on motor control and trunk stabilization aligns with emerging evidence supporting non-aerobic therapeutic strategies for LC patients, particularly those with postural instability or autonomic dysfunction.

Miana et al. demonstrated that a personalized supine motor control exercise program significantly reduced fatigue and body fat percentages in women with LC, without exacerbating symptoms [31]. These findings underscore the importance of tailoring exercise interventions to individual symptom profiles, especially in patients with PESE. Concerns about PESE in LC rehabilitation have prompted calls for symptom-titrated protocols to prevent adverse responses [50].

The reduction in the MFIS scores, particularly in physical and psychosocial domains, supports the effectiveness of non-aerobic, core-stabilization exercises in mitigating fatigue in LC without triggering PESE [51]. This is especially relevant given the high prevalence of autonomic dysfunction and chronotropic incompetence in LC patients, which can impair cardiovascular responses to exercise and exacerbate symptoms. These findings suggest that motor control-based interventions can be safely implemented even in patients with autonomic dysfunction, offering a viable strategy for improving functional outcomes [52,53].

Recent studies have demonstrated that MFIS scores correlate strongly with other quality of life metrics, such as the EQ-VAS and EQ-5D-5L, reinforcing their utility in evaluating therapeutic outcomes. For instance, Cozma et al. [54] found that persistent fatigue in LC patients was significantly associated with reduced scores in the Nottingham Health Profile (NHP), particularly in the domains of energy, physical activity, and pain, which were positively correlated with MFIS scores [55]. These findings underscore the central role of fatigue in shaping the overall health perception and functional capacity of LC patients. The MFIS allows for the detection of improvements in fatigue-related quality of life that may not be captured by changes in body composition or aerobic capacity. In our study, despite the absence of significant morphological changes, participants reported substantial improvements in MFIS scores, suggesting that neuromuscular adaptations and enhanced motor control were the primary drivers of recovery. This aligns with evidence from Miana et al. [31], who reported significant reductions in MFIS scores following a personalized, non-aerobic motor control exercise program in women with LC. The psychosocial domain of the MFIS, though comprising only two items, is particularly sensitive to changes in emotional and social functioning. Improvements in this domain may reflect enhanced coping strategies, reduced isolation, and increased confidence in physical capabilities—factors that are critical in the long-term rehabilitation of LC patients. Given the complex interplay between physical symptoms and psychological distress in LC, the MFIS provides a comprehensive framework for monitoring patient progress and tailoring interventions accordingly [56]. The observed improvements in MFIS scores following core-stabilization exercises highlight the potential of non-aerobic, motor control-based interventions to enhance functional outcomes and overall well-being in LC rehabilitation, even in the absence of aerobic conditioning.

The improvement in EQ-VAS scores and the trend toward better outcomes in the “usual activities” dimension of the EQ-5D-5L further highlight the functional relevance of the intervention [57]. The results found in our study are consistent with those of Smith et al. [58] and Griffiths et al. [27], who reported enhanced HRQoL following structured rehabilitation programs in individuals with LC. These findings emphasize the importance of integrating patient-centered outcome measures in LC rehabilitation research. The observed improvements in EQ-VAS and trends in functional domains suggest that core-focused interventions may contribute meaningfully to perceived health recovery, even in the absence of significant changes in traditional clinical parameters. However, the observed improvement in EQ-VAS without corresponding changes in EQ-5D-5L domains should be interpreted with caution. EQ-VAS reflects a global, subjective perception of health, which may respond to transient or psychosocial factors, whereas EQ-5D-5L domains capture specific functional aspects that require more substantial changes to show variation. This dissociation indicates that perceived overall health improvement does not necessarily translate into measurable gains in individual dimensions, underscoring the need for careful interpretation of these results.

This study presents several notable strengths that enhance its scientific and clinical relevance. Firstly, it addresses a critical gap in LC rehabilitation by evaluating a non-aerobic, core-focused intervention designed to mitigate fatigue—a symptom consistently reported as one of the most disabling in LC populations. The use of the MFIS and EQ-5D-5L/EQ-VAS instruments allowed for a multidimensional assessment of both fatigue and HRQoL, providing a comprehensive view of patient outcomes. The intervention was grounded in motor control principles and implemented under real-world conditions, supervised by licensed physiotherapists, which enhances its ecological validity and potential for clinical translation. The study design incorporated a pragmatic approach to group allocation, enabling participation from individuals with varying levels of availability and commitment. This reflects the realities of LC rehabilitation, where symptom variability and fluctuating energy levels often limit adherence to rigid protocols. The intervention’s safety profile—marked by the absence of adverse events and the avoidance of PESE—further supports its feasibility in vulnerable populations.

However, several limitations must be acknowledged: (i) the quasi-experimental design and non-randomized group allocation may introduce selection bias and limit causal inference; (ii) all participants were vaccinated, which restricts generalizability to unvaccinated individuals. Nevertheless, given the high vaccination coverage in Spain during recruitment, the cohort is locally representative of health-service-engaged LC patients. Future randomized trials will include or stratify by vaccination status to quantify potential effect modification on rehabilitation outcomes. In addition to this, (iii) the study population was limited to women with LC, which, while addressing a frequently underrepresented demographic, may not reflect the broader LC population, including men and individuals with different symptom profiles. Moreover, (iv) the small and uneven sample size significantly reduces the statistical validity of the findings and limits the generalizability of conclusions to other LC populations, and (v) there is an absence of objective physiological markers such as inflammatory cytokines, autonomic function tests, or neuroimaging data, which could have elucidated the mechanisms underlying the observed improvements. (vi) The pilot design also did not allow for the reporting of effect sizes alongside *p*-values, which would have provided a better quantification of the magnitude of observed changes, and (vii) the lack of objective physiological indicators represents an important limitation of this study. No exercise testing, heart rate variability (HRV) analysis, autonomic function assessment, or inflammatory biomarkers were included, which restricts the ability to explore underlying mechanisms and confirm physiological adaptations. While the primary aim was to evaluate patient-reported outcomes under pragmatic conditions, future research should incorporate these objective measures to provide a more comprehensive understanding of the intervention’s effects. (viii) Furthermore, while the MFIS and EQ-5D-5L are validated instruments, their sensitivity to subtle changes in LC symptomatology may be limited, particularly in fluctuating conditions. In addition, (ix) the intervention focused exclusively on core-stabilization exercises, without integrating other potentially beneficial modalities such as respiratory training, cognitive rehabilitation, or nutritional support, and (x) the study did not include long-term follow-up, which limits the ability to assess the sustainability of the observed improvements over time. (xi) Future research should include standardized tools to rule out alternative causes of fatigue, and (xii), additionally, the absence of randomization and blinding in group allocation represents a relevant methodological limitation, as it may introduce selection bias. Participants assigned to the intervention group were those able to commit to two weekly sessions, which could imply that individuals with better health status or higher motivation were overrepresented. Although a pragmatic approach was adopted to reflect real-world rehabilitation conditions, we acknowledge that this strategy reduces the internal validity of the study. More representative cohorts are needed to confirm these findings and to explore subgroup effects, including gender and age, in future studies.

Future research should prioritize randomized controlled trials with larger and more diverse cohorts to strengthen the generalizability of the findings. Incorporating long-term follow-up is essential to determine whether the observed improvements in fatigue and quality of life are sustained over time. Additionally, studies should include objective physiological markers—such as autonomic function tests and inflammatory biomarkers—to better understand the mechanisms underlying recovery. Multimodal interventions that combine motor control exercises with respiratory training and cognitive rehabilitation may offer synergistic benefits and warrant exploration. Finally, investigating dose–response relationships will help optimize exercise prescription and tailor rehabilitation strategies for individuals with LC.

## 5. Conclusions

This study demonstrates that a 12-week core-focused plank exercise program can effectively reduce fatigue and improve self-perceived health status in women with LC. While no significant changes were observed in body composition parameters, participants in the intervention group experienced meaningful improvements in the physical and psychosocial domains of fatigue, as measured by the MFIS, and in overall health perception, as reflected by the EQ-VAS scores. This program may serve as a safe rehabilitation option for individuals with PESE.

However, these conclusions must be interpreted with caution due to the small and uneven sample size and the absence of randomization, which limit statistical validity and generalizability. Further research into larger, more diverse cohorts and appropriate adjustments for age, gender, and comorbidities are required before extrapolating these findings to the broader LC population.

These findings support the use of non-aerobic, motor control-based exercise interventions as a safe and beneficial strategy for managing persistent fatigue and enhancing quality of life in patients with LC.

## Figures and Tables

**Table 1:** Baseline characteristics of LC participants.

			Total (n = 39)	Intervention (n = 20)	Control (n = 19)	*p* Value
Years, mean (SD)			46.64 (5.90)	45.50 (7.09)	47.84 (4.19)	0.220
Months with COVID-19 symptoms, mean (SD)			19.74 (7.73)	18.75 (9.75)	20.83 (4.64)	0.415
Initial COVID-19 symptoms	Hospital admission	Yes, n (%)	10 (25.6)	5 (25.0)	5 (26.5)	0.925
		No, n (%)	29 (74.4)	15 (75.0)	14 (73.5)	
	Pneumonia	Yes, n (%)	16 (41.0)	6 (30.0)	10 (52.6)	0.151
		No, n (%)	23 (59.0)	14 (70.0)	9 (47.4)	
	Emergency	Yes, n (%)	28 (71.8)	12 (60.0)	16 (84.2)	0.093
		No, n (%)	11 (28.2)	8 (40.0)	3 (15.8)	
	Reinfection	Yes, n (%)	5 (12.8)	3 (15.0)	2 (10.5)	0.676
		No, n (%)	34 (87.2)	17 (85.0)	17 (89.5)	
	COVID-19 previous diseases	Yes, n (%)	24 (61.5)	14 (70.0)	10 (52.6)	0.265
		No, n (%)	15 (38.5)	6 (30.0)	9 (47.4)	
	Previous surgery	Yes, n (%)	29 (74.4)	14 (70.0)	15 (78.9)	0.522
		No, n (%)	10 (25.6)	6 (30.0)	4 (21.1)	
	Previous respiratory disease	Yes, n (%)	12 (30.8)	8 (40.0)	4 (21.1)	0.200
		No, n (%)	27 (69.2)	12 (60.0)	15 (78.9)	
Fatigue	Persistent fatigue	Yes, n (%)	37 (94.9)	18 (90.0)	19 (100.0)	0.157
		No, n (%)	2 (5.1)	2 (10.0)	0 (0.0)	
Cognitive impairment	Brain fog	Yes, n (%)	26 (66.7)	15 (75.0)	11 (57.9)	0.257
		No, n (%)	13 (33.3)	5 (25.0)	8 (42.1)	
	Concentration problems	Yes, n (%)	33 (84.6)	18 (90.0)	15 (78.9)	0.339
		No, n (%)	6 (15.4)	2 (10.0)	4 (21.1)	
	Memory problems	Yes, n (%)	24 (61.5)	14 (70.0)	10 (52.6)	0.265
		No, n (%)	15 (38.5)	6 (30.0)	9 (47.4)	
Respiratory symptoms	Dyspnea	Yes, n (%)	20 (51.3)	13 (65.0)	7 (36.8)	0.071
		No, n (%)	19 (48.7)	7 (35.0)	12 (63.2)	
	Chronic cough	Yes, n (%)	0 (0.0)	0 (0.0)	0 (0.0)	1.000
		No, n (%)	39 (100.0)	20 (100.0)	19 (100.0)	
	Musculoskeletal pain	Yes, n (%)	31 (79.5)	18 (90.0)	13 (68.4)	0.095
		No, n (%)	8 (20.5)	2 (10.0)	6 (31.6)	
	Sleep disorders	Yes, n (%)	6 (15.4)	4 (20.0)	2 (10.5)	0.412
		No, n (%)	33 (84.6)	16 (80.0)	17 (89.5)	
	Anxiety/depression	Yes, n (%)	3 (7.7)	2 (10.0)	1 (5.3)	0.579
		No, n (%)	36 (92.3)	18 (90.0)	18 (94.7)	

SD—standard deviation.

## Data Availability

The data presented in this study are available on request from the corresponding author. The data are not publicly available due to legal restrictions.
